# Anti-Aquaporin-1 Autoantibodies in Patients with Neuromyelitis Optica Spectrum Disorders

**DOI:** 10.1371/journal.pone.0074773

**Published:** 2013-09-23

**Authors:** John S. Tzartos, Christos Stergiou, Konstantinos Kilidireas, Paraskevi Zisimopoulou, Thomas Thomaidis, Socrates J. Tzartos

**Affiliations:** 1 Department of Biochemistry, Hellenic Pasteur Institute, Athens, Greece; 2 Department of Neurology, General Hospital “Red Cross", Athens, Greece; 3 Department of Neurology, Aeginition Hospital, School of Medicine, National and Kapodistrian University, Athens, Greece; 4 Department of Pharmacy, University of Patras, Patras, Greece; Kyushu University, Japan

## Abstract

Autoantibodies against aquaporin-4 (AQP4), a water channel in CNS astrocytes, are detected in ∼50–80% of patients with neuromyelitis optica spectrum disorders (NMOsd), characterized by longitudinally extensive transverse myelitis (LETM) and/or optic neuritis. Although these autoantibodies present an invaluable biomarker for NMOsd and for the differential diagnosis of multiple sclerosis (MS), diagnosis of anti-AQP4-seronegative NMOsd remains challenging. We hypothesized that seronegative NMOsd patients might have autoantibodies against aquaporin-1 (AQP1), another water channel in CNS astrocytes. We initially developed a radioimmunoprecipitation assay to search for anti-AQP1 antibodies in sera from 632 individuals. Anti-AQP1 or anti-AQP4 autoantibodies were detected in 16.7% and 12%, respectively, of 348 patients with suspected NMOsd. Anti-AQP1 specificity was confirmed by competition, protein immunoblotting and ELISA assays, whereas epitope localization was studied by immunoadsorption on intact cells expressing AQP1 and peptide mapping experiments. Most anti-AQP1 autoantibodies were of the complement-activating IgG1 subclass and the majority bound to the extracellular domain of AQP1, suggesting a possible pathogenic role. Five out of 42 MS patients had anti-AQP1 antibodies, but 2 of them also had spinal cord lesions, while the anti-AQP1 antibodies in the other 3 bound to the cytoplasmic domain of AQP1. Anti-AQP1 antibodies were not detected in 100 healthy individuals or 142 patients with non-demyelinating neuroimmune diseases. Analysis of 17 anti-AQP1+/anti-AQP4- patients with suspected NMOsd showed that 5 had NMO and 11 had LETM. 12/17 of these sera bound predominantly to the extracellular AQP1 loop-Α. Overall, we found that anti-AQP1 autoantibodies are present in a subgroup of patients with chronic demyelination in the CNS and similarities with anti-AQP4-seronegative NMOsd, offering a novel potential biomarker for CNS demyelination disorders.

## Introduction

Patients suffering from chronic inflammatory demyelinating diseases of the central nervous system (CNS) present a variable disease course, pathology, and response to treatment [1]. As a consequence, specific biomarkers are needed to classify these patients and adopt the most appropriate treatment. Currently, autoantibodies to aquaporin-4 (AQP4), a water channel abundantly expressed in CNS astrocytes, are a widely accepted biomarker in patients suffering from neuromyelitis optica spectrum disorders (NMOsd). The NMOsd group of demyelinating disorders includes the classic neuromyelitis optica (NMO), with major characteristics longitudinally extensive transverse myelitis (LETM) and optic neuritis [2], only LETM, only recurrent optic neuritis and related disorders [3]. In a high proportion of NMOsd patients, brain lesions are also present [4], [5], thus complicating diagnosis, and the detection of anti-AQP4 antibodies is therefore vital for the early differentiation of NMO from MS [6] and for appropriate treatment [7]. Unfortunately, about 20–50% of NMOsd patients are seronegative for anti-AQP4 autoantibodies [8]–[10].

AQP4 belongs to the large AQP family with 13 members (AQP0-12), which form tetramers in the cell membrane, each monomer acting as a water channel [11]. AQPs have been identified in several mammalian tissues [12]. Several reports have shown that, in addition to AQP4, human CNS astrocytes also abundantly express AQP1 on their surface [13]–[17]. Specifically, it has been shown that AQP1 is highly expressed in areas prone to develop NMO lesions (spinal cord, optic nerves and brain white matter) [17]. Moreover, both AQP1 and AQP4 are overexpressed in the brain in some neurological diseases, such as MS, compared to “normal” brains [13], possibly for maintenance of water homeostasis. We therefore wondered whether patients with NMOsd, but seronegative for anti-AQP4 antibodies, had antibodies to AQP1. NMO-related autoantibodies (named NMO-IgG) were initially identified using an immunofluorescence assay in which serum from NMO patients was applied to mouse brain sections, resulting in a characteristic pattern [18]. Subsequently, it was found that NMO-IgG were anti-AQP4 antibodies [19]. Interestingly, anti-AQP1 antibodies would not be detected in the usual immunofluorescence assay for NMO-IgG using rodent brain tissue because AQP1, although expressed in rodent brain, it is not detected in their astrocytes [16], [20]–[22] and pre-treatment of serum with liver powder (known to contain AQP1 [23]), used as a blocking agent in this assay, selectively removes anti-AQP1 antibodies, as shown in this manuscript.

Here, we screened sera from 348 patients with suspected NMOsd and found that a higher percentage contained anti-AQP1 antibodies (∼17%) than anti-AQP4 antibodies (12%). Anti-AQP1 antibodies were not detected in 242 control sera. The clinical and radiological characteristics for 17 anti-AQP1-seropositive patients with suspected NMOsd suggest remarkable similarities between anti-AQP1-seropositive patients and anti-AQP4-seronegative NMOsd patients.

## Materials and Methods

### Serum samples

Serum samples were obtained from 348 patients whose doctors had requested testing for anti-AQP4 autoantibodies. Additionally, sera from 42 patients diagnosed with MS (fulfilling the McDonald 2010 MS criteria [24]), 142 sera from patients with other neuroimmune diseases, and 100 sera from healthy individuals were used as controls (total 632 serum samples). The 142 patients with other neuroimmune diseases consisted of 101 patients with anti-acetylcholine receptor antibody-positive myasthenia gravis (MG), 30 patients with anti-MuSK antibody-positive MG, 5 patients with antibodies against Ca^2+^ channels (responsible for Lambert-Eaton myasthenic syndrome), and 6 patients with other autoantibodies involved in neuroimmunological diseases (antibodies against K^+^-channels, gangliosides GM2, GQ1b or GD1A, or MAG and YO antigens).

### Ethics Statement

Sera were used after the approval of the Bioethics Committee of the Hellenic Pasteur Institute (last approval ref. no 1288/2012) and the participants provided written informed consent to participate in this study in a form approved by the above Bioethics Committee.

### Plasmid constructs and AQP proteins

pEGFP-N1 was purchased from Clontech Laboratories, CA, USA. The pCMV6-AQP1-tGFP (AQP1-GFP) and pCMV6-AQP4-M23-tGFP (AQP4-GFP) were both purchased from OriGene Technologies Inc. (Rockville, MD, USA) as ready to use plasmids. The cDNAs encoding human AQP1 and human AQP4 were also subcloned into pPICZα and pPIC9, respectively (Life Technologies, Invitrogen), in order to be expressed in yeast (with 6xHis and 8xHis tags for AQP1 and AQP4, respectively) and were purified by Ni-NTA chromatography and gel filtration. In addition, a commercially available fusion protein of human recombinant AQP1 fused with a glutathione-S-transferase tag (AQP1-GST) was expressed in the wheat germ cell-free protein expression system and affinity purified on immobilized glutathione (Novus Biologicals, Cambridge, UK). The yeast-expressed AQP1 was generated at a later stage and was used on selected sera (including all sera in Table 1) to confirm the findings.

**Table 1:** Laboratory and clinical data for anti-AQP1 seropositive patients.

Patient no.	Sex	Age at onset (years)	Disease duration (years)	Anti-AQP4 Abs^a^	Anti-AQP1 titer (nM)^b^	Abs bound to intact AQP1-expressing cells (%+SD)^c^ Titer (nM)^d^		Predominant epitope^e^	MRI and clinical data
1	F	34	8	no	1.9	NT	NT	N-term	LETM and optic neuritis (NMO)
2	F	18	3	no	2.7	5±2	0.1	-	LETM and optic neuritis (NMO)
3	F	47	2	no	2.5	96±6	2.4	Loop-C	LETM and optic neuritis (NMO)
4	M	23	20	no	3.7	88±10	3.3	Loop-A	LETM and optic neuritis (NMO)
5	F	39	28	no	3.9	79±3	3.1	Loop-A	LETM and optic neuritis (NMO)
6	F	27	1	no	1.6	ΝΤ	NT	N-term	LETM
7	F	38	2	no	2.7	13±3	0.4	Loop-B	LETM
8	F	34	2	no	1.8	30±3	0.5	Loop-E	LETM
9	F	28	6	no	2.6	99±2	2.6	Loop-A	LETM
10	F	39	12	no	2.0	99±6	2.0	Loop-A	LETM
11	M	34	3	no	5.8	88±1	5.1	Loop-A	LETM
12	M	33	9	no	4.2	16±5	0.7	Loop-A	LETM
13	F	40	10	no	10.5	92±6	9.7	Loop-A	LETM
14	F	23	14	no	2.8	82±5	2.3	Loop-A	LETM
15	M	30	18	no	2.6	8±10	0.2	Loop-B	LETM.Hodgkin lymphoma
16	M	43	20	no	2.7	77+10	2.1	Loop-A	LETM and brain lesions (fulfilled Barkhof criteria)
17	M	39	1	no	2.5	93±2	2.3	Loop-A	Transverse myelitis. Kidney neoplasm
18	F	42	5	no	4.0	100±1	4.0	Loop-E	MS with predominant spinal cord lesions
19	F	44	25	no	3.8	92±6	3.5	Loop-A	MS with predominant spinal cord lesions and axonal neuropathy. Mammary neoplasm
20	F	17	26	no	3.9	0±1	0.0	Loop-B	MS
21	F	18	19	no	3.0	6±1	0.2	Loop-B	MS
22	F	35	8	no	2.6	2±4	0.1	Loop-B	MS

F, female; LETM, longitudinally extensive transverse myelitis; M, male; MS, multiple sclerosis; NMO, neuromyelitis optica; NT, not tested.

a All 22 patients tested negative for serum anti-AQP-4 autoantibodies in the two-step RIPA and confirmed by both a commercial and an in-house CBA.

b The anti-AQP1 positivity of the sera was also confirmed by the more sensitive two-step RIPA for anti-AQP1 antibodies (not shown).

c According to the Methods and Figure 8.

d Calculated by multiplying anti-AQP1 titers (in 6^th^ column) by the percentages of the extracellularly binding antibodies (in 7^th^ column).

e Binding of sera to synthetic peptides corresponding to AQP1 segments (from Figure 9B). Underlined loops are extracellular.

### Maintenance and transfection of human embryonic kidney cells (HEK 293)

The HEK293 cell line was grown in Dulbecco’s modified eagle’s medium (DMEM; Life Technologies, Gibco) supplemented with 10% FCS and 100 units/ml each of penicillin G and streptomycin (Life Technologies, Invitrogen) at 37°C in an atmosphere of 5% CO_2_. HEK-293 cells were seeded and allowed to grow for at least 24 hours on 10 cm plates when used for competition and cell-immunoadsorption assays. Cells were transiently transfected with Lipofectamine 2000 (Life Technologies, Invitrogen) at 40–50% confluency.

### Detection of antibody binding to AQPs by RIPA and two-step RIPA

RIPA. Human AQPs were indirectly labeled with ^125^I by incubating 0.25 nmol of AQP with 5 nmol of NHS-PEO_4_-Biotin on ice for 2 h, then incubating approximately 0.1 µg of biotinylated AQP for 1 h on ice with 50,000 cpm of ^125^I-streptavidin radioiodinated by the chloramine-T method to a specific activity of 1600–1870 Ci/mmol. For the RIPA, 5 µl or less of serum was incubated for 2 h at 25°C with 50 µl of phosphate-buffered saline (PBS) containing 0.5% Triton X-100, pH 7.4 (assay buffer) and 0.1 µg of the indirectly labeled AQP, then 50 µl of goat anti-human immunoglobulin (Ig) was added and the mixture incubated for 2 h at 25°C and centrifuged, and the pellet washed twice with 1 ml of assay buffer and the radioactivity in the pellet measured in a gamma-counter.

The average value for sera from 30 healthy human individuals plus 3 standard deviations, SD (∼250+3×35 = ∼355 cpm) was arbitrarily multiplied by 1.4. This value (∼500 cpm) was considered to be the cut-off for a positive result, and the average value plus 3 SD arbitrary multiplied by 1.1 (∼390 cpm) was taken as the cut-off for an “ambiguous” result; the cut-offs correspond to antibody titers of 2.5 and 1.4 nM, respectively.

Two-step RIPA. In addition to the above RIPA, a more sensitive two-step RIPA used to detect anti-AQP4 autoantibodies, and in selected cases of low anti-AQP1 titer sera, was also used to confirm the anti-AQP1 positivity of the sera. This assay was based on a method involving semi-purification of the specific autoantibodies from a large volume of serum (∼150 µl) by non-stringent affinity chromatography and their use in the standard RIPA (described in Trakas et al. 2011 [25]).

### Detection of antibody binding to AQPs by ELISA

Antibody binding to yeast-expressed AQP1, was also studied by an ELISA assay. 96-well plates were coated with 0.5 µg peptide, or BSA as control, (in 50 µL of 0.01 M carbonate buffer, pH 9.6) per well. After overnight incubation at 4°C, plates were washed 3 times with PBS/0.05% Tween-20 and once with PBS and the remaining sites were blocked by incubation with Superblock blocking buffer (Thermo Scientific, Rockford, USA) for 1 h at room temperature. After 3 washes of the plates with PBS/0.05% Tween 20, and once with PBS, sera were added at a 1/100 dilution in PBS/2% BSA and the plates incubated overnight at 4°C. After washing the plates as above, horseradish peroxidase-conjugated rabbit anti-human IgG antibodies diluted 1/5000 in 2% BSA/PBS were added and the plates incubated for 1 h at room temperature, washed as above, and incubated for 15 min at room temperature with 3,3′,5,5′-tetramethylbenzidine (TMB). Color development was quantified at 450 nm after stopping the reaction with 0.2 M sulfuric acid. Values were considered positive when they were 50% or more higher than the mean OD_450_ for normal control sera, a value which, using serum samples from 44 healthy controls, was higher than the mean OD_450_ of the controls plus 3SD. Specifically, the average OD_450_ for these control sera for AQP1 was 0.24 with SD 0.03, and thus the cut-off for positive values was 0.36. The O.D.450 by all tested sera for plated BSA was 0.23±0.06.

### Effect of guinea pig liver powder on the AQP1-RIPA

Five microliter samples of test sera were diluted in 100 µl of 0.2% bovine serum albumin (BSA) in PBS and pretreated with 20 mg of guinea pig liver powder (Rockland Inc, Gilbertsville, PA.) for 1 h at room temperature. After centrifugation, the supernatants were tested by AQP1-RIPA and AQP4-RIPA and the results compared to those obtained using untreated sera.

### Competition RIPA experiments

To test whether the antibodies bound to the AQP1-GST fusion protein or to ^125^I-streptavidin, 10 AQP1-antibody positive sera and negative controls were tested for direct binding to the same amount of ^125^I-streptavidin. To test whether the antibodies bound to the AQP1 moiety of the AQP1-GST fusion protein, 10 µl of sera positive for anti-AQP1 autoantibodies was preincubated for 3 h at 4°C with excess GST (1.6 µg) immobilized on Sepharose-glutathione beads (GE Healthcare), then 5 µl of the treated samples was tested in the RIPA using ^125^I-streptavidin-labeled AQP1-GST.

Competition tests in which commercial AQP1-GST and AQP1 expressed in-house in HEK293 cells or yeast competed for binding to serum antibodies were performed as follows: Five microliter samples of anti-AQP1 antibody-containing sera were preincubated for 3 h at 4ΊC (i) with extracts of nontransfected HEK293 cells or HEK293 cells transfected with human AQP1-GFP prepared by solubilizing 500,000 cells in 500 µl of homogenization buffer (100 mM NaCl, 100 mM KCl, 5 mM EDTA, 5 mM EGTA, 50 mM Tris pH 7.5, and 1% Triton X-100)] or (ii) with 10 µl of assay buffer containing 0.2 µg of either AQP1 or AQP4 expressed in yeast cells and purified, or with 0.2 µg of BSA as a control. These mixtures were then tested in the regular RIPA using indirectly labeled commercial AQP1-GST.

### Detection of antibody binding to AQP1 by Western blotting

Samples (0.45 µg of protein per lane) of yeast-expressed AQP1 or a control protein (MuSK) were electrophoresed on a 4–12% gradient SDS-polyacrylamide gel and transferred onto nitrocellulose membranes. The membranes were then incubated for 1 h at 25°C with 3% BSA in PBS, then for 2 h at 4°C with a commercial rabbit anti-AQP1 polyclonal antibody (AB3272; Millipore, Temecula, CA), anti-AQP1-positive sera, or sera from healthy individuals, all diluted (30-500 times) in 0.2% BSA in PBS. The membranes were then washed and incubated for 90 min at 4°C with a 1∶1000 dilution of horseradish peroxidase (HRP)-conjugated anti-rabbit or HRP-conjugated anti-human antibody, washed with PBS, and were with the chromomeric substrate 3,3’-diaminobenzidine (DAB) to visualize antibody binding.

### Investigation of cross-reactivity between anti-AQP1 and anti-AQP4 antibodies

Double-positive sera were depleted of one type of antibody as follows: 0.2 µg of either AQP1 or AQP4 (or control BSA) was immobilized in each ELISA well, then 150 µl of 2% BSA in PBS containing a suitable volume of test serum was added and the mixture incubated for 16 h at 4°C. The supernatants were then tested in parallel RIPAs for the presence of anti-AQP1 and anti-AQP4 antibodies to determine the level of the remaining antibody against each antigen.

### Determination of anti-AQP1 antibody class and subclass

The Ig subclass of the anti-AQP1 autoantibodies was investigated using the RIPA with the following modification. After the incubation of labeled antigen with the serum sample, 5 µl of sheep anti-human IgG subclass or sheep anti-human IgM antibodies (1 mg/ml; Binding Site, Birmingham, UK) was added and the mixture incubated overnight at 4°C, then 20 µl of rabbit anti-sheep Ig antiserum (Sigma-Aldrich, Munich, Germany) was added and the mixture incubated for 2 h at room temperature, then the pellets were washed and their radioactivity counted.

### Measurement of antibodies binding to the extracellular region of AQP1

HEK293 cells were transfected with AQP1-GFP, AQP4-GFP and pEGFP-N1. The culture medium used after transfection was supplemented with 10^−7^ M secretin (Bachem, Bubendorf, Switzerland) which induces surface expression of AQP1 [4] whereas in parallel transfections with the same plasmids no secretin was added. The cells were collected 48 h after transfection, washed twice with PBS, and counted. Five or 10 microliter samples of anti-AQP1 antibody-containing sera were preincubated for 2 h at room temperature with 50,000–500,000 intact transfected HEK293 cells in 50 µl of PBS buffer, then the mixtures were centrifuged and the supernatants tested in the regular RIPA to determine the titre of unbound antibodies.

### Peptides and peptide mapping experiments

Six synthetic peptides corresponding to the extracellular and cytoplasmic segments of human AQP1 were synthesized and HPLC-purified (>80% purity) by JPT Peptide Technologies GmbH (Berlin). These corresponded to the extracellular segments 36–49 (including Loop-A), 116–137 (including Loop-C), and 183–188/199–210 (including Loop-E) and the intracellular segments 1–22 (N-terminus), 81–100 (Loop-B), and 248–270 (C-terminus).

Antibody binding to the peptides was studied by ELISA. Ninety-six-well plates were coated by overnight incubation at 4°C with 0.2 µg peptide, or BSA as control, in 50 µL of 0.01 M carbonate buffer, pH 9.6, per well, then were washed 3 times with PBS/0.05% Tween-20 and once with PBS, and the remaining sites blocked by incubation for 1 h at room temperature with 4% BSA in PBS. The following steps were as described above with the ELISA with intact AQP1. Values were considered positive when they were 50% or more higher than the mean OD_450_ for normal control sera, a value which, using serum samples from 12 healthy controls, was always higher than the mean OD_450_ of the controls plus 3SD. In practice, the average OD_450_ for these 12 control sera for any peptide (and for BSA instead of peptide) was between 0.25–0.29 (many more sera from healthy controls subsequently tested gave very similar values), the SDs were between 0.03–0.05, and the cut-off for positive values was ∼0.45.

### Cell based assays (CBA)

In house CBA for anti-AQP4 antibodies was performed according to Leite et al. [9]. In addition, a commercial AQP4-CBA kit (Euroimmun, Luebeck, Germany) was also used according to the manufacturer’s instructions.

## Results

### Anti-AQP1 autoantibodies are present in sera of patients with suspected NMOsd at a similar frequency to anti-AQP4 autoantibodies

**Figure 1:**
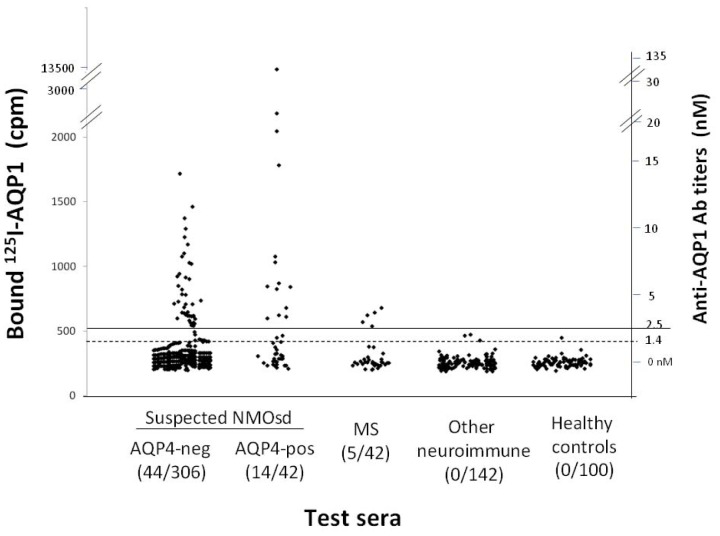
Measurement of anti-AQP1 antibodies in the sera of different patient groups and healthy controls by RIPA. The serum samples are (left to right) from patients with suspected NMOsd without (AQP4-neg) or with (AQP4-pos) anti-AQP4 antibodies, MS, other neuroimmune diseases (131/142 with MG), or healthy controls. The numbers in parenthesis below the group are the number of anti-AQP1 antibody-positive sera and the total number of test sera for each group of patients. The dashed and solid horizontal lines denote the cut-off values for ambiguous and positive titers. The left and right y axis show, respectively, the precipitated cpm and the estimated antibody titer.

We developed a RIPA for anti-AQP1 autoantibodies using, as labeled antigen, a human AQP1-GST fusion protein biotinylated and indirectly labeled with ^125^I-streptavidin. This RIPA for anti-AQP1 antibodies was used to examine sera from 348 patients whose doctors had requested screening for anti-AQP4 antibodies. These sera had previously been tested for anti-AQP4 antibodies using the very sensitive two-step RIPA and 42 had been classed as anti-AQP4 antibody-positive. In the present study, 44 of the 306 anti-AQP4 antibody-negative (14.4%) were found to be positive for anti-AQP1 antibodies, as were 14 of the 42 anti-AQP4 antibody-positive sera (33%) (Figure 1). Overall, 16.7% of the tested sera (58/348) were anti-AQP1 antibody-positive. The titer range of anti-AQP1 antibodies in our anti-AQP1-positive sera was similar to that of the anti-AQP4 titers in our anti-AQP4-positive sera; specifically, after excluding the top 10% of titers in each group which caused very high standard deviations, the average values of the remaining 52 anti-AQP1-positive and 40 anti-AQP4-positive sera were 5.3±2.2 nM and 6.3±10.2 nM (not shown), respectively. 142 sera from patients with autoantibodies related to non-demyelinated neuroimmune diseases (the 131 with MG) and 100 sera from healthy controls were negative (Figure 1). However, 5 of 42 sera from patients diagnosed with MS had low anti-AQP1 antibody titers, but, as described below, these patients either had concurrent spinal cord lesions or their antibodies did not bind to intact AQP1-expressing cells but rather bound to cytoplasmic epitopes. The female/male ratio of the 44 patients with exclusively anti-AQP1 antibodies was 1.9:1.

**Figure 2:**
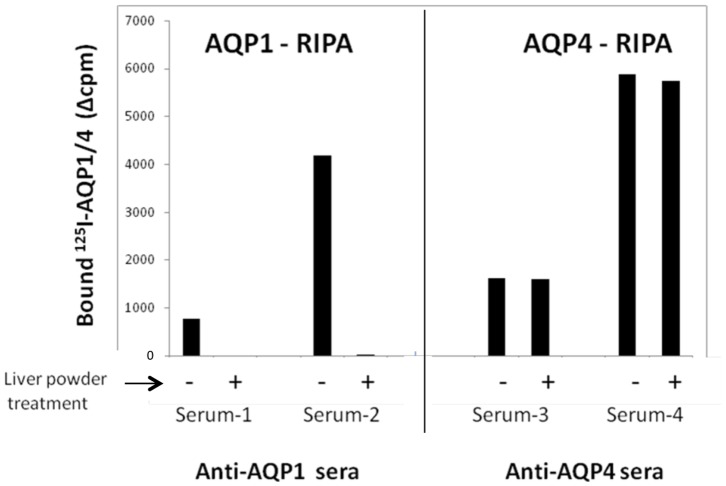
Liver powder removes anti-AQP1 antibodies. Two exclusively anti-AQP1-positive and two exclusively anti-AQP4-positive serum samples were pretreated with guinea pig liver powder, then the supernatants were tested by RIPA using indirectly radiolabeled AQP1 (left panel) or AQP4 (right panel). Key: +, pretreated serum; -, untreated serum.

The above RIPA assay and the identified anti-AQP1-positive sera were then used to investigate whether guinea pig liver powder, which is routinely used as a blocking agent in immunofluorescence assays for the detection of NMO-IgG, removed anti-AQP1 antibodies from the anti-AQP1-antibody containing sera, thus preventing their detection in these assays. When anti-AQP1 and anti-AQP4-positive sera were pretreated with guinea pig liver powder and the supernatants assessed by RIPA, the liver powder completely removed the anti-AQP1 antibodies, but had no effect on the anti-AQP4 antibodies (Figure 2), apparently because liver contains AQP1 [23]. Thus, anti-AQP1 autoantibodies would escape detection in the immunofluorescence assay for NMO-IgG.

**Figure 3:**
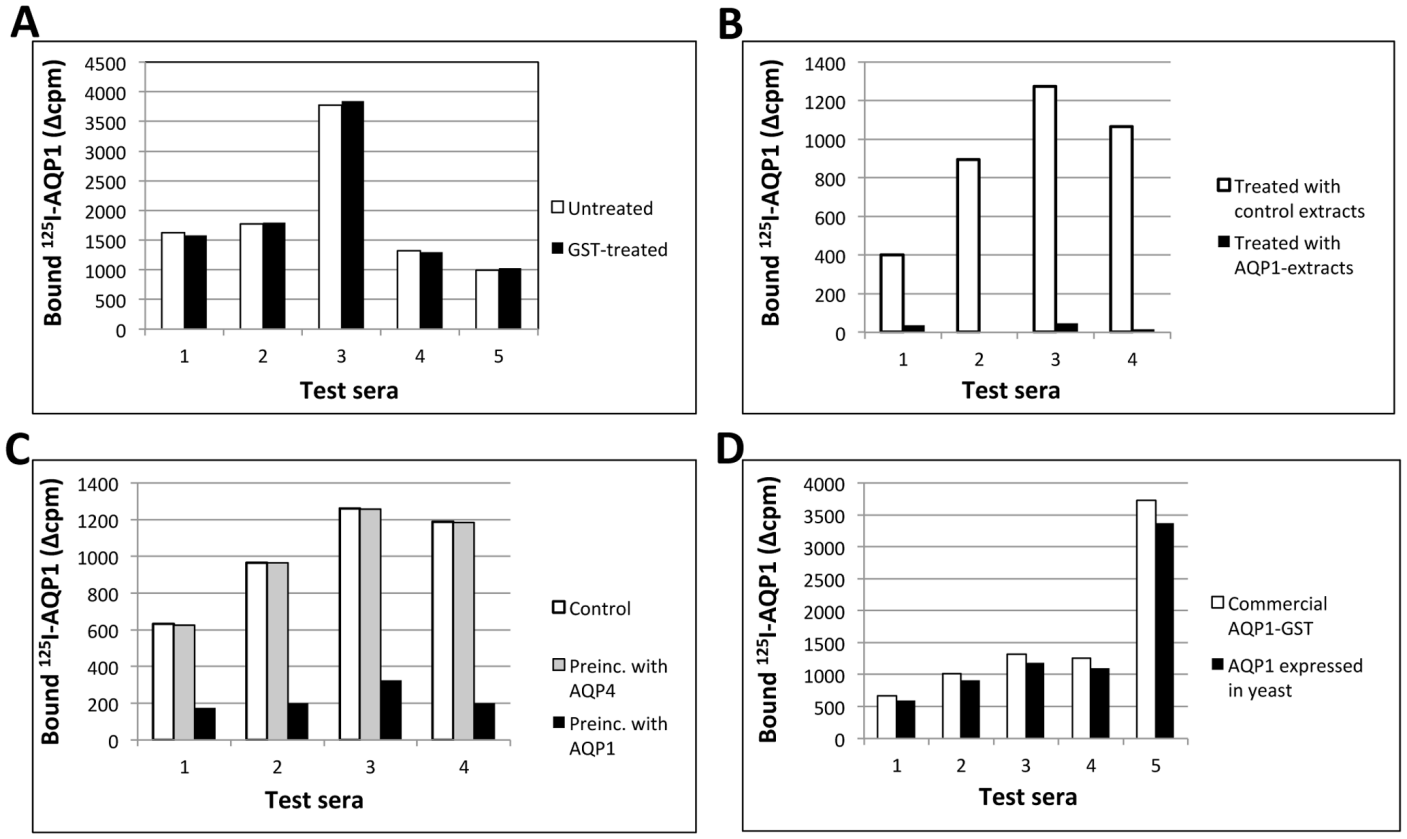
Anti-AQP1 specificity of the identified autoantibodies. (A) Patient’s autoantibodies recognize the AQP1 moiety of AQP1-GST. Five sera that had tested positive for binding to the AQP1-GST fusion protein were preincubated with an excess of GST immobilized on Sepharose-glutathione beads, then were tested by RIPA using ^125^I-streptavidin labeled AQP1-GST. (B) Binding of anti-AQP1 autoantibodies is specifically inhibited by an extract from AQP1-expressing HEK293 cells, but not control HEK293 cells. Four anti-AQP1-positive sera were preincubated with extracts prepared from either EGFP-transfected or AQP1-GFP-transfected HEK293 cells, then were tested by RIPA for binding to the commercial AQP1 preparation. (C) Binding of anti-AQP1 autoantibodies is specifically inhibited by yeast-expressed human AQP1. Four exclusively anti-AQP1-positive sera were preincubated with human AQP1 or AQP4 that had been expressed in yeast and purified or with BSA as control, then were tested by RIPA using ^125^I-streptavidin-labeled commercial AQP1-GST fusion protein. (D) AQP1 autoantibody binding is independent of the source of AQP1. Both the commercial AQP1-GST fusion protein and the in house AQP1 purified from yeast were biotinylated, indirectly labeled by preincubation with ^125^I-streptavidin, and used in the RIPA. Five anti-AQP1-positive sera and one serum sample from a healthy control (HC) were tested.

Several experiments confirmed the specificity of the anti-AQP1 RIPA assay. Initially, we confirmed that the antibodies bound exclusively to the AQP1 moiety of the AQP1-GST-^125^I-streptavidin complex by showing that “anti-AQP1 antibodies” in 10 positive sera did not bind to either ^125^I-streptavidin (not shown) nor the GST moiety of the AQP1-GST fusion protein (Figure 3A). We then checked whether antibodies binding to the human AQP1-GST preparation, expressed in a cell-free system, also bind to human AQP1 expressed in alternative systems, in the following experiments: First, binding of antibodies to commercial AQP1-GST labeled with ^125^I-streptavidin was inhibited by pre-incubation of the sera with extracts from AQP1-GFP expressing HEK293 cells, but not with extracts from nontransfected HEK293 cells (Figure 3B) or with purified yeast-expressed AQP1, but not with purified yeast-expressed AQP4 (Figure 3C). Second, when yeast-expressed AQP1 was used as the labeled antigen in the RIPA, the results were very similar to those obtained using the commercial AQP1 (Figure 3D). These experiments show that antibody binding is independent of the source of AQP1 antigen used.

**Figure 4:**
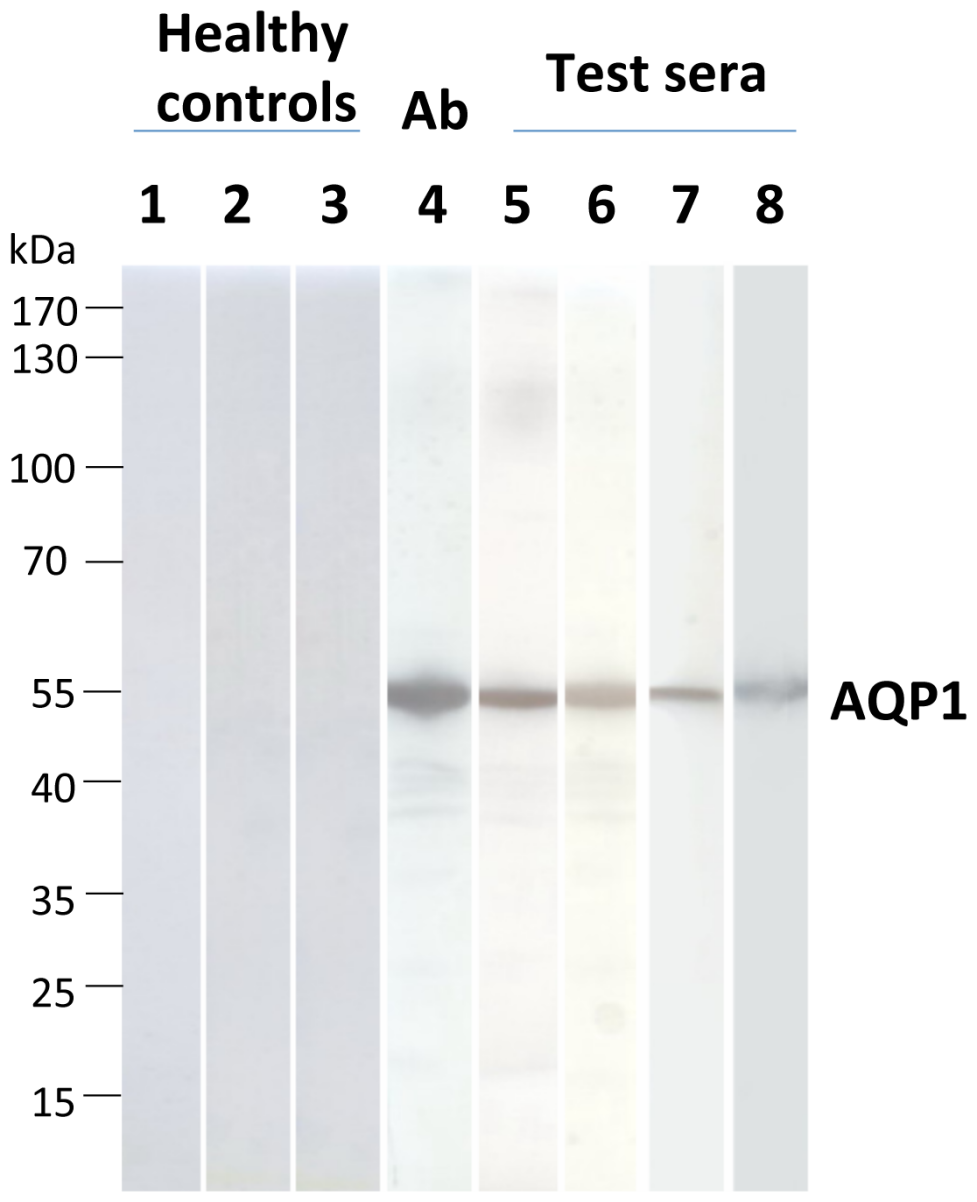
Detection of binding of anti-AQP1 antibody to denatured AQP1 by Western blotting. Yeast-expressed AQP1 was electrophoresed and transferred onto nitrocellulose membranes, which were then incubated with the test sera (see Methods). Lanes 1-3: Three sera from healthy controls at dilutions 1/250, 1/60, and 1/30, respectively; lane 4: commercial rabbit anti-AQP1 antibody; lanes 5-8: representative anti-AQP1-positive sera with titers of 132, 25, 10.5, and 5.8 nM, at dilutions of 1/500, 1/250, 1/30, and 1/30, respectively. None of the test anti-AQP1 sera bound to the control protein MuSK (not shown).

The RIPA results were then confirmed by Western blotting. All 4 anti-AQP1 antibody-containing sera tested bound to electrophoresed denatured yeast-expressed AQP1 (Figure 4). This approach provides an alternative assay for anti-AQP1 autoantibodies and shows that serum antibodies also bind to denatured AQP1.

**Figure 5.**
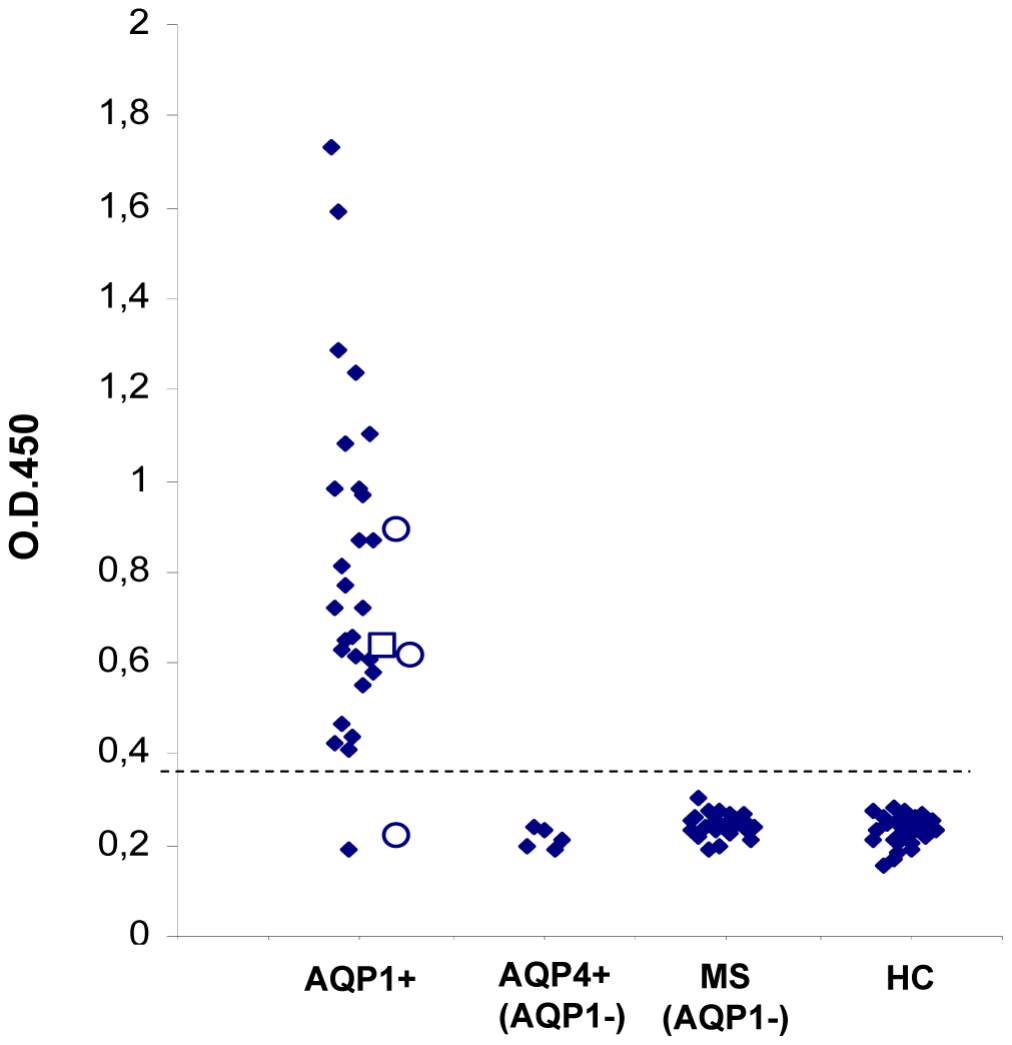
Detection of anti-AQP1 antibodies by an ELISA with immobilized purified human yeast-expressed AQP1. Sera previously tested by RIPA for AQP1 antibodies were tested for binding to immobilized AQP1 by ELISA. First column contains 31 sera found positive by the RIPA (including a double-positive anti-AQP1/AQP4, empty square, and 3 sera from anti-AQP1-positive MS patients, empty circles). The following 3 columns contain 5 anti-AQP4-positive/anti-AQP1-negative sera, 30 sera from anti-AQP1-negative MS patients and 44 sera from healthy controls). The dashed horizontal line denotes the cut-off (O.D.450: 0.36) for positive values.

Finally, we developed an ELISA with immobilized purified yeast-expressed AQP1 to further confirm the RIPA results and possibly provide an easier method for future routine diagnosis. We tested three serum dilutions (1/25, 1/100 and 1/500) of which the 1/100 dilution gave the best results and was adopted for the following experiments. Figure 5 shows that there is very good correlation between the results of RIPA and ELISA: all tested 79 sera previously found negative for AQP1 antibodies by the RIPA (44 from healthy controls, 5 from anti-AQP4+/AQP1- sera and 30 from anti-AQP1-negative MS patients) were clearly negative, whereas 29/31 sera previously found anti-AQP1 positive by the RIPA (including one double-positive and two MS sera) were also positive by the ELISA. Only 2/31 sera (i.e. 6.5%) were positive by RIPA and negative by ELISA.

### Anti-AQP1 and anti-AQP4 autoantibodies are specific for the corresponding antigen

**Figure 6:**
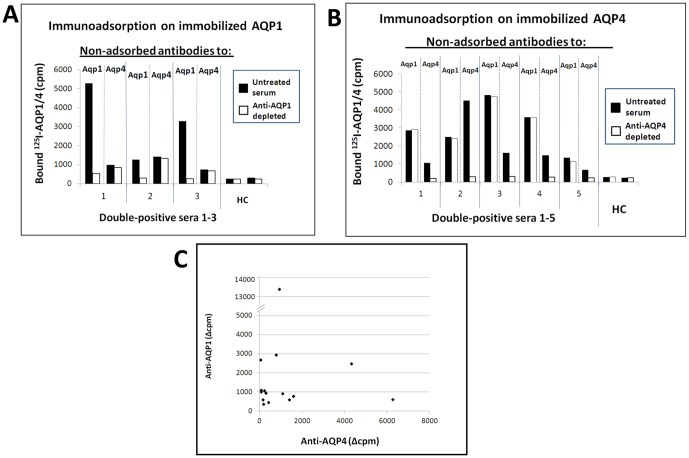
Anti-AQP1 antibodies and anti-AQP4 antibodies do not bind to the other antigen. (A and B). Search for cross-reactivity of anti-AQP1 and anti-AQP4 antibodies with AQP4 and AQP1, respectively. Three (A) or five (B) double-positive serum samples were incubated with immobilized AQP1 (A) or AQP4 (B) to immunoadsorb the corresponding antibodies, then were tested by RIPA for binding to ^125^I-labeled AQP1 or AQP4 (white bars) in parallel with the untreated sera (black bars). HC, healthy control. (C). Lack of correlation of the amount of radiolabeled AQP1 precipitated by anti-AQP1 antibodies (y axis) or AQP4 (x axis) precipitated anti-AQP4 antibodies by double-positive sera in identical regular RIPAs performed using the two labeled antigens.

We then tested whether the double-positivity (anti-AQP1 and anti-AQP4) seen with some sera was due to cross-reactivity by preincubating selected double-positive sera with one of the antigens immobilized on ELISA plates, then examining whether the unbound material was able to bind to either of the antigens. The results showed that immobilized AQP1 removed the anti-AQP1 antibodies, but not the anti-AQP4 ones (Figure 6A), while immobilized AQP4 removed only the anti-AQP4-antibodies (Figure 6B). We subsequently tested whether there was a correlation between the concentrations of the two antibodies in the double-positive sera, but no such correlation was detected (Figure 6C). However, sera with a relatively high titer of antibodies for one AQP were more likely to be double-positive than lower titer sera (not shown). In conclusion, the double-positive sera contain two sets of antibodies, each specific for one AQP.

### The vast majority of anti-AQP1 antibodies are IgG1

**Figure 7:**
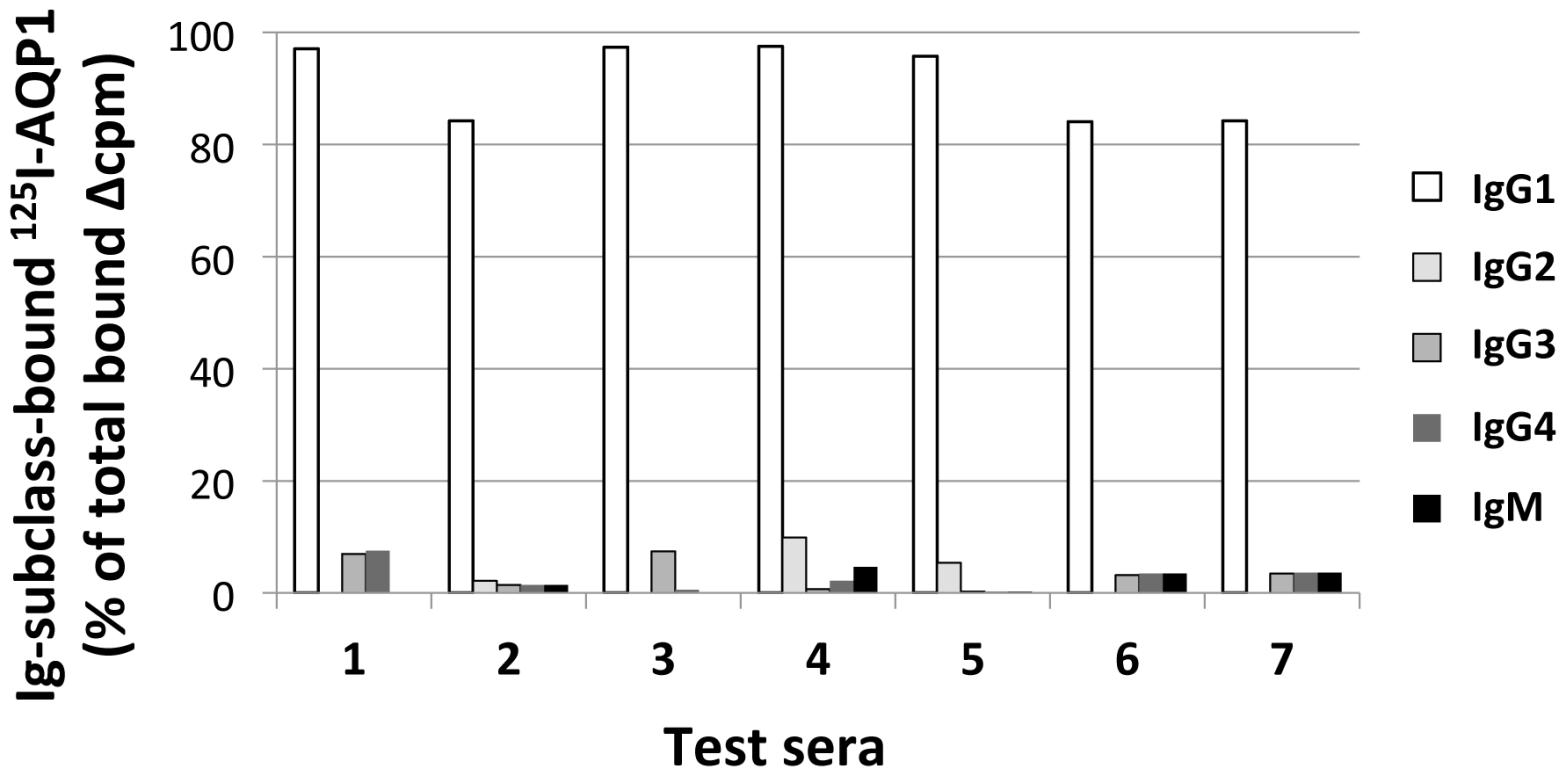
Ig class and IgG subclass of the anti-AQP1 antibodies. The Ig class and IgG subclass of the anti-AQP1 antibodies from 7 positive human sera was tested by RIPA using anti-class/subclass second antibodies. Sera no. 1-4 are from patients 11-14 in Table 1 and sera no. 5-7 are from the remaining anti-AQP1 antibody-positive sera.

We then examined the Ig class and subclass of the anti-AQP1 antibodies in sera from 7 patients (4 sera from patients no. 11-14 in Table 1 with available clinical data, and the other 3 randomly selected from the remaining anti-AQP1 antibody-positive sera). All 7 sera showed a marked predominance of the complement-activating IgG1 subclass (Figure 7).

### Most anti-AQP1 antibodies of NMO-suspected patients bind to the extracellular region of AQP1

**Figure 8:**
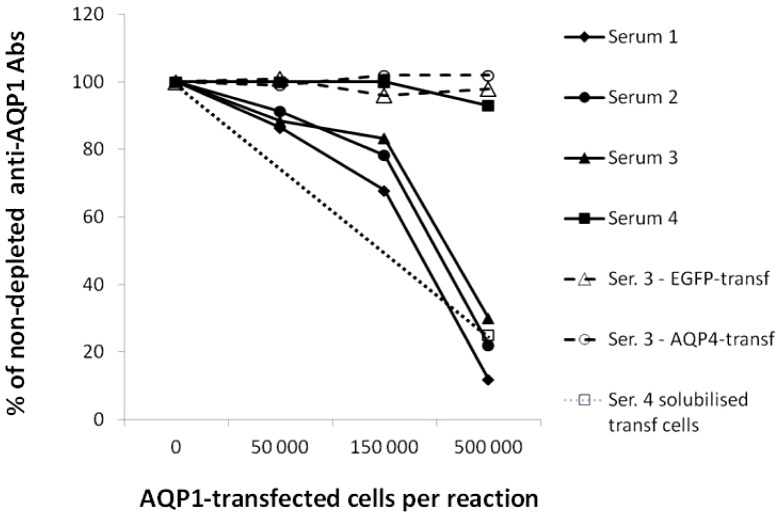
Determination of the percentage of antibodies directed against the extracellular domain of membrane-embedded AQP1. Four anti-AQP1-positive sera were left untreated or were preincubated with increasing numbers of AQP1-GFP- (filled symbols) or EGFP-transfected (shown only for serum 3; empty triangle) HEK293 cells treated with secretin to increase surface expression of AQP1; the untreated and treated samples were tested in the usual RIPA for anti-AQP1 antibodies. The sera were also treated with AQP4-transfected HEK293 cells (shown only for serum 3; open circle). Serum 4 was also treated with an extract of AQP1-transfected HEK293 cells (open square).

We then examined whether the anti-AQP1 antibodies could bind to intact HEK293 cells expressing AQP1. We used a quantitative approach to determine the fraction of antibodies that bind to the extracellular region of AQP1. This involved a combined immunoadsorption with RIPA experiment in which the test sera were preincubated with intact AQP1-GFP-transfected or EGFP-transfected HEK293 cells followed by RIPA on the depleted sera. To enhance surface expression of AQP1, the cells were pretreated with the hormone secretin [26]. As shown in Figure 8, secretin-treated AQP1-GFP transfected HEK293 cells efficiently removed the anti-AQP1 antibodies from 3 of the 4 test sera (sera no. 1-3), while EGFP-transfected secretin-treated cells or AQP4-transfected cells had no effect (only representative results are shown in Figure 8). Serum no. 4 was practically not depleted of its antibodies by the intact AQP1-transfected cells; however its preincubation with detergent-solubilised AQP1-transfected cells efficiently inhibited antibody binding in the RIPA suggesting that most anti-AQP1 antibodies of this serum bind to the cytoplasmic domain of AQP1. As shown in Figure 8, 500.000 AQP1-transfected secretin-treated cells were necessary and sufficient for efficient immunoadsorption of the anti-AQP1 antibodies. In the absence of secretin, immunoadsorption of anti-AQP1 antibodies by 500.000 cells was insignificant (∼0-10%) but it was efficient when we used 4 million cells per reaction (∼50-75% immunoadsorption for 3 tested sera; not shown). AQP4-transfected cells (but not AQP1-transfected or EGFP-transfected cells) were very efficient in removing anti-AQP4 antibodies in similar assays for the detection of anti-AQP4 antibodies (in fact, they were ∼10 times more efficient than the AQP1-transfected secretin-treated cells since only 50,000 AQP4-transfected cells (without secretin-treatment) were sufficient to deplete the relevant sera; data not shown). These results suggest that antibodies to both the extracellular and the cytoplasmic domains of AQP1 may exist in anti-AQP1-positive sera.

### Epitope mapping of the anti-AQP1 antibodies

**Figure 9:**
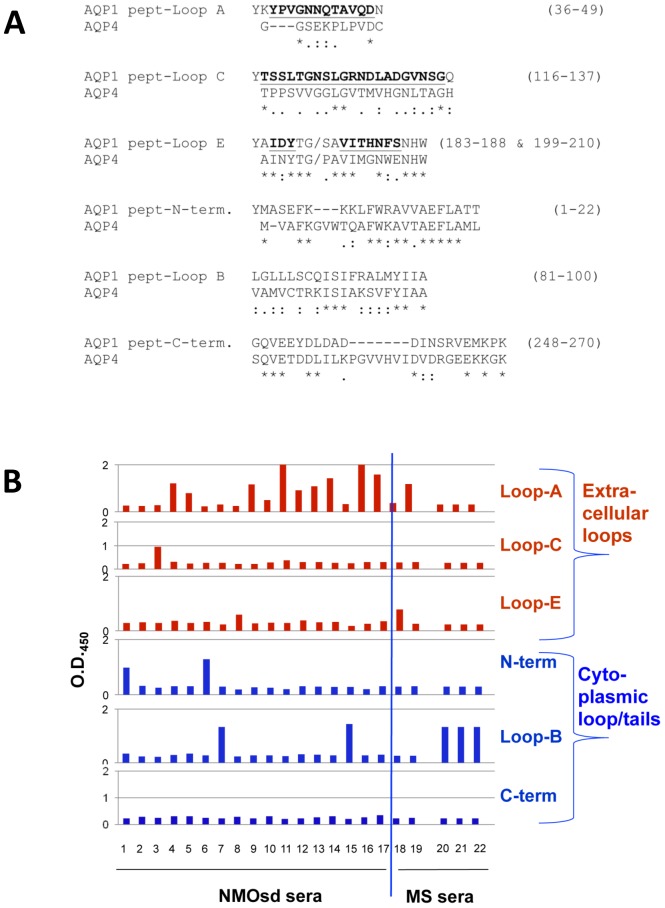
Anti-AQP1 antibody binding to synthetic peptides corresponding to AQP1 extracellular and cytoplasmic domains. **A.** Synthetic peptides corresponding to human AQP1 extracellular loops A (residues 36–49), C (116–137), and E (183–188, 199–210) or to the intracellular N-terminus (1–22), loop B (81–100), and the C-terminus (248–270) are shown (from Uniprot/Swissprot entry P29972, for the human AQP1). Extracellular residues are shown in bold and underlined. Loop-E is formed by two segments separated by a 14 residue gap; in “pept-Loop-E”, 10 of these 14 residues (between TG and SE) were omitted (marked by:..). In the first 4 peptides, an extra tyrosine residue was added to the N-terminus for future radioiodination studies. Below the AQP1 peptide sequences are shown the corresponding AQP4 sequences; (*), identical amino acid residues; (:) and (.), homologous residues. **B.** Peptide mapping with anti-AQP1 positive sera. 22 sera from patients with known clinical characteristics (17 NMOsd and 5 MS) were tested for binding to 6 synthetic peptides corresponding to the 3 extracellular loops (loops A, C and E) and to the 3 cytoplasmic segments (N-terminal, Loop-B and C-terminal) by an ELISA assay. Positive values are considered those above O.D._450_ 0.45 (see Methods). It is shown that most NMOsd sera bind to the Loop-A peptide.

To confirm the above results using a different approach and to locate more precisely the epitopes recognized by the anti-AQP1 antibodies, we used ELISA to test the binding of several anti-AQP1 sera to synthetic peptides corresponding to the 3 extracellular loops (Loops A, C, and E) and the 3 main cytoplasmic segments (Loop B and the N-terminal and C-terminal) of AQP1 (Figure 9A). Figure 9B shows the results for the binding of 22 sera from patients with known clinical characteristics as described in the next paragraph. Interestingly, with one exception (serum no 2, which did not bind to any peptide), all bound almost exclusively to a single peptide; specifically, 14 sera bound to extracellular peptides (11 to Loop-A, 1 to Loop-C, and 2 to Loop-E) and 7 to cytoplasmic peptides (5 to Loop-B and 2 to the N-terminal). No antibodies bound to the peptide corresponding to the C-terminal of AQP1. Loop A, which is the most antigenic, presents quite low identity (14%) and homology (43%) with the corresponding AQP4 sequences, whereas Loop E presents high similarity with the homologous sequence of AQP4 (67% identity and 89% homology); nevertheless, the two sera which bound to peptide Loop E (sera no. 8 and 18 in Figure 9B) did not bind to intact AQP4. As described below, peptide mapping correlated very well with the above immunoadsorption/RIPA results.

### Retrospective analysis of MRI and clinical data for 22 anti-AQP1-seropositive patients

Magnetic resonance imaging (MRI) and clinical data were available for 22 anti-AQP1-seropositive, anti-AQP4-seronegative patients, consisting of 17 with suspected NMOsd and 5 MS patients (shown in Figure 1). In addition to the AQP1-RIPA, 19 of these sera were also tested by ELISA with intact AQP1 (included in Figure 5); only two of the 19 sera (sera no 10 and 21 in Table 1) did not bind in the ELISA. Anti-AQP4 seronegativity was determined by a two-step RIPA and confirmed by two CBA assays (in house according to [9] and commercial; see Methods), all found anti-AQP4 negative. Table 1 shows that 16 of the 17 patients with suspected NMOsd had LETM, five of which also had optic neuritis (i.e. typical NMO) and one had only transverse myelitis. Fifteen sera from these 17 patients were also tested, in addition to RIPA, for antibodies binding to intact AQP1-expressing cells using immunoadsorption on intact AQP1-transfected HEK293 cells followed by RIPA (as described in the previous chapter and in Figure 8). The percentage of antibodies in each serum binding to the extracellular region of AQP1 was 79–99% in 10 sera, 13–30% in 3, and <10% in 2 sera (7^th^ column in Table 1). These 22 sera were also tested for binding to synthetic peptides corresponding to the 6 AQP1 segments (Figure 9, described above). Interestingly, whether or not a high percentage of the antibodies in a serum sample bound to the intact AQP1-expressing cells correlated very well with whether or not they bound to an extracellular peptide (compare columns 7 and 9 in Table 1), further confirming the extracellular location of the antibody binding sites for most of these sera.

Of the 5 anti-AQP1-positive MS patients (no. 18–22 in Table 1), the first two (no. 18 and 19) also had predominant spinal cord lesions (though not LETM) and most (92–100%) of their anti-AQP1 antibodies bound to intact AQP1-expressing HEK293 cells. Interestingly, the anti-AQP1 antibodies in the remaining 3 patients (no. 20–22) with classic MS practically did not bind to the intact AQP1-expressing cells (0–6%) and bound to the cytoplasmic AQP1 Loop-B. Finally, 3 of the 22 anti-AQP1 positive patients also had neoplasms (nephroma, non-Hodgkin lymphoma, or mammary cancer).

## Discussion

Many NMOsd patients are seronegative for anti-AQP4 antibodies [8]–[10], and, although AQP4 loss is usually observed in NMO brain lesions, no such loss has been observed in some NMO patients [27]. Recently, antibodies to the myelin-oligodendrocyte glycoprotein (MOG) have also been detected in seronegative NMO patients, however in limited cases (4/27) [28]. AQP1 might be a major candidate autoantigen for anti-AQP4-seronegative NMOsd, since, like AQP4, it is abundantly expressed in human astrocytes [13]–[16]. Interestingly, a recent study by Misu et al [17] showed that, in some NMO lesions, astrocyte AQP1 cell surface expression was selectively reduced or AQP1 was found in astrocytic intracellular granules, results similar to those observed for AQP4. However, in this study, we showed that anti-AQP1 autoantibodies would not be detected in the classic assay for NMO-IgG antibodies (Figure 2). Consequently, different approaches were necessary for the detection of anti-AQP1 autoantibodies.

We initially developed a RIPA for anti-AQP1 antibodies and screened sera from patients with suspected NMOsd. Interestingly, anti-AQP1 antibodies were more frequent than anti-AQP4 antibodies, with 16.7% of the 348 tested sera being anti-AQP1-positive and only 12% anti-AQP4-positive (Figure 1). Overall, the anti-AQP-seropositive patients were doubled, from 12% anti-AQP4 to 24.7% anti-AQPs (taking into account that 4% were double positive). It should be noted that, generally, only a small fraction (∼9–12%) of patients referred for laboratory diagnosis of NMOsd are found to be anti-AQP4-positive [29]. The low female/male ratio of the anti-AQP1-seropositive patients (1.9:1) is identical to that determined for anti-AQP4 antibody-negative NMO patients in a recent large multicenter study [30], but much lower than that of 10.4:1 for the anti-AQP4-positive patients, determined in the same study or the 4.2:1 ratio we found in our anti-AQP4-seropositive patients. The relevance of the anti-AQP1 autoantibodies to NMOsd was supported by their absence in sera from 100 healthy individuals and 142 patients with non-demyelinating neuroimmune diseases (131 with MG) (Figure 1). The specificity of the anti-AQP1 antibodies in patients’ sera was also confirmed by several competition experiments (Figure 3) and by the independent determination of autoantibody binding by Western blotting (Figure 4). Finally, although in general RIPAs are more sensitive and specific than ELISAs, because ELISAs are preferable in routine diagnosis we also developed a simple and sensitive ELISA with intact affinity-purified AQP1 which overall agrees well with the RIPA results (Figure 5). Only 2/31 (6.5%) sera previously found anti-AQP1 positive by the RIPA could not bind AQP1 in the ELISA. Therefore, AQP1-ELISA should prove useful in future routine diagnosis. These findings suggest that anti-AQP1 autoantibodies are at least as frequent as anti-AQP4 autoantibodies and thus anti-AQP1-seropositive patients may form a major subset of anti-AQP4-seronegative NMOsd patients.

Despite the fact that the two AQPs share considerable amino acid sequence identity (∼51%), the two sets of antibodies in the double-positive sera showed no cross-reactivity (Figure 6A,B) or correlation of titers (Figure 6C). Nevertheless, the high frequency of double-positives (24% of the anti-AQP1-positives) and the observation that sera with a higher antibody titer for either AQP have a greater chance of being double-positive (data not shown) might indicate a related, or interdependent, immune system triggering mechanism. Since several human astrocytes express both AQPs [13], epitope spreading across the AQPs in the double-positive patients is a possibility.

Since the extracellular versus cytoplasmic location of the binding site for anti-AQP1 antibodies is potentially important for their potential pathogenic significance, we then developed assays to determine the location of binding of the anti-AQP1 antibodies. Despite many efforts and moderate success, we failed to develop a reproducible and reliable CBA using AQP1-transfected HEK293 cells, the main reason apparently being the observed low concentration of cell surface-expressed AQP1. Use of secretin increased surface expression of AQP1, but this was still much lower than the observed surface expression of AQP4 and it also dramatically increased background immunofluorescence. We therefore developed two alternative assays which quantitatively measure the location of the antibody binding sites on AQP1: (a) immunoadsorption of the serum antibodies on secretin-treated intact AQP1-transfected cells followed by RIPA of the unbound antibodies and (b) an ELISA to test serum binding to 3 synthetic peptides corresponding to the 3 extracellular loops and 3 peptides corresponding to most of the cytoplasmic domain of AQP1. The results of the two assays correlated very well, revealing which sera contained antibodies that bound mainly to the extracellular or the cytoplasmic domain of AQP1. In addition, peptide mapping revealed that the predominant extracellular epitope(s) of the tested sera were on Loop-A of AQP1.

The observed very good correlation between the immunoadsorption/RIPA and peptide mapping results was not necessarily expected, as often only a small minority of the antibodies to a protein antigen bind to linear peptides. Thus, in preliminary experiments, we tested whether the antibodies that bound to the synthetic peptides were the majority or a small minority; we performed competition RIPA experiments, in which 7 test sera were preincubated with 0.1–1.0 µg of individual peptides or with peptide mixtures (extracellular or cytoplasmic peptide groups) before incubation with labeled AQP1 in the regular RIPA. The results showed that, for each serum, ≥0.5 µg of the specific peptide, but not of the other peptides, inhibited antibody binding to AQP1 by >50% (not shown), demonstrating that the majority of these anti-AQP1 antibodies could bind to linear sequences of AQP1, and thus the correlation between the results for the two approaches (immunoadsorption on intact cells and peptide mapping) was well justified. Nevertheless, because often small peptides also bind antibodies to proteins with small homologies with the specific antigen, in order to minimize false-positive results, anti-AQP1 presence should be detected with the use of intact AQP1 (e.g. by RIPA or ELISA with intact AQP1) whereas peptide-ELISA should only be used for epitope mapping of the anti-AQP1 positive sera.

Retrospective analysis of the available MRI and clinical data was possible for 22 patients positive only for anti-AQP1 antibodies (17 suspected NMOsd patients among the seropositives in the 1^st^ column of Figure 1 and the 5 seropositive MS patients in the 3^rd^ column of Figure 1). This analysis showed that 19 patients had spinal cord lesions (Table 1). Specifically, the vast majority (16) had LETM (5 also had optic neuritis, i.e. typical NMO), one had only transverse myelitis, and 2 were diagnosed with MS but also had a predominant spinal cord lesion load. Furthermore using intact AQP1-expressing cells we showed that the majority of the anti-AQP1 antibodies of the tested sera from the patients with spinal cord lesions of Table 1 bound to the extracellular domain of AQP1 (Table 1, column 7; average: 66.5%): 79–99% in 10 sera, 13–30% in 3, and only 2 sera had <10% of their antibodies to the extracellular domain of AQP1. In addition, peptide mapping experiments confirmed that the targets for these antibodies were predominantly extracellular and further restricted the major antigenic region to extracellular Loop-A (Figure 9B and Table 1). The cell-damaging factor in the patients with low concentrations of antibodies to the extracellular side of AQP1 may be different, as in the double-seronegative NMOsd patients, although these patients may also produce small quantities of antibodies to the extracellular side of AQP1, which were already immobilized on their tissue AQP1. Overall, these data strongly suggest that anti-AQP1 autoantibodies are a potential biomarker for NMOsd among anti-AQP4-seronegative patients.

The remaining 3 of the 22 patients of Table 1 (no. 20–22) had classic MS. However, the presence of anti-AQP1 autoantibodies in patients diagnosed with MS is not surprising, since NMO-IgG and anti-AQP4 autoantibodies have been detected in patients diagnosed with MS (∼4% [18] to 25% [8]) and it was recently shown that sera from many MS patients bind to synthetic peptides corresponding to cytoplasmic epitopes of AQP4 [31]. Interestingly, almost none (0–6%) of the anti-AQP1 antibodies in the sera of the 3 patients with classic MS were able to bind to AQP1 on intact cells (Table 1, 7^th^ column), nor to the extracellular loop peptides, suggesting that anti-AQP1 antibodies in classic MS may bind to the cytoplasmic domain of AQP1.

Importantly, 3 out of the 22 patients of Table 1 also had neoplasms, suggesting a paraneoplastic factor, similar to findings in NMO patients with anti-AQP4 antibodies [32]. Although the small numbers prohibit reliable conclusions for the frequency of neoplasms in anti-AQP1-seropositive patients, they suggest that this frequency may be roughly similar with that of the anti-AQP4-seropositive patients [32]. It is still unknown whether the anti-AQP1 autoantibodies play any pathogenic role in NMOsd. Since, in contrast to the situation in human astrocytes [13]–[16], AQP1 is not expressed on rodent astrocytes [16], [19]–[21], rodents cannot be used as a model to study the pathogenic effect of anti-AQP1 autoantibodies on astrocytes and their role in NMOsd; therefore alternative models must be developed to investigate the possible pathogenic role of these antibodies. Nevertheless, the roughly similar range of anti-AQP1 and anti-AQP4 antibody titers (8,7±17,3 nM versus 7,8±12,5 nM, respectively) the similar IgG1 subclass predominance, and most antibodies binding to the extracellular domain of AQP1, suggest that a similar level of pathogenic potency of these antibodies is likely (possibly inducing local damage of astrocytes by a complement-mediated mechanism). In conclusion, we detected the presence of autoantibodies specific for AQP1 mainly in anti-AQP4-seronegative NMOsd patients, providing a promising diagnostic biomarker for this disease subtype, and observed similarities and differences to anti-AQP4-seropositive NMOsd. The low female-to-male ratio, the absence of coexisting autoimmune diseases (e.g. MG), and the predominance of LETM (with a low incidence of optic neuritis) in the anti-AQP1-seropositive NMOsd patients are similar to the characteristics of anti-AQP4-seronegative NMOsd [30], [33]. These correlations and the high frequency of autoantibodies to the extracellular domain of AQP1 in patients with suspected NMOsd suggest that anti-AQP1-seropositivity may characterize a major subset of anti-AQP4-seronegative NMOsd patients.
